# Flourishing and Posttraumatic Growth. An Empirical Take on Ancient Wisdoms

**DOI:** 10.1007/s10728-016-0318-2

**Published:** 2016-03-25

**Authors:** Hugh Middleton

**Affiliations:** School of Sociology and Social Policy, University of Nottingham, Law and Social Sciences Building, Nottingham, NG7 2RD UK; Nottinghamshire Healthcare NHS Trust, Nottingham, UK

**Keywords:** Consumerism, Eudaimonism, Flourishing, Hedonism, Post-traumatic growth, Redemption, Sick role, Suffering, Well-being

## Abstract

Considerations of well-being or flourishing include Maslow’s and Rogers’ concepts of self-actualisation and actualising tendency. Recent empirical findings suggest that only a modest proportion of the population might be considered to be flourishing. Separate findings focused upon the nature and determinants of post-traumatic growth identify it as comparable to flourishing, and facilitated by supported accommodation to the trauma. This can be understood as reflecting self-actualisation. Empirical findings such as these provide ontological stability to a set of phenomena that share much with ancient teachings extolling redemption through suffering. This framework challenges conventional healthcare policies and practices, but in ways that offer insights into how patient-centred approaches to chronic illness and disability might be better conceived and enabled. It also throws into doubt the rectitude of an economic model built around services and products designed to provide easy access to sources of immediate gratification.

## Introduction

Although the poetry might not have been so evocative, Cohen could have used “languishing” rather than “suffering” in the last line of this stanza. From the research psychologist’s perspective flourishing entered their lexicon as an antonym to languishing. The terms are used in a consideration of mental health and well-being using data captured by the 1995 Midlife Development in the United States (MIDUS) survey [17]. However, by using “suffering” and making reference to entrapment by “pleasures” Cohen resonates with much more ancient traditions of thought. A common feature of all the major religions; Buddhism, Christianity, Hinduism, Islam and Judaism, is teachings drawing attention to the possibility of improved well-being as a result of renouncing pleasures, “sin”, attachments or mundane habits. Other ancient traditions have parallels. The notion of redemption through suffering and self-denial, and with it improved longer term well-being is an established feature of how the experience of being human is understood. The study of post-traumatic growth can be considered an application of scientific method and frameworks to this, with all the strengths and shortcomings associated with the application of science to human affairs. This paper traces some of the more recent history of that process. It summarises how the phenomenon of post-traumatic growth is currently understood, and what might be learned from it for health and other aspects of public policy if human flourishing is to be enabled. In relation to healthcare policy and practice, the dynamics of post-traumatic growth stand in contrast to those of the traditional sick role, which advocates dependency and professionalised interventions. Patient-centred care and particularly provision for those with persistent difficulties also challenge that orthodoxy and the paper considers how lessons concerning flourishing and post-traumatic growth might be applied in that context.

## Background

Landmarks of thinking about psychological well-being during the early twentieth century include Maslow’s concept of self-actualisation perhaps originally attributable to Goldstein [8], Rogers’ actualising tendency and Jahoda’s reflections upon the nature of mental health.

Maslow drew attention to the notion of an individual as an essentially creative entity moving towards fulfilment in the form of self-actualization. This has much in common with perspectives upon the human condition which give voice, in one form or another, to the notion of spirituality. His well-known hierarchy of needs outlines individual fulfilment as progress through the satisfaction of more basic and physiological needs such as food, drink and sexual appetite, needs for safety, needs for love, acceptance and belongingness, needs for esteem, approval and recognition to self-actualization [22]. An essential and inherently autonomous self is implicit in notions of self-actualization, personal growth and development and the effects of influences that might hinder them. “Self” cannot be understood or conceptualized without reference to ways in which it is constructed and experienced through interactions with other individuals and the wider landscape of human experience and consciousness. Maslow described self-actualized people as those who:

… listen to their own voices; they take responsibility; they are honest; and they work hard. They find out who they are and what they are, not only in terms of their mission in life, but also in terms of the way their feet hurt when they wear such and such a pair of shoes and whether they do or do not like eggplant … All this is what the real self means (Maslow [23] p. 49).

Other contemporary and subsequent practitioners and theorists have added to and adapted this framework, most notably Carl Rogers who is associated with approaches to therapy which broadly speaking, advocate the enabling of actualizing tendency: human beings’ natural motivation towards constructive growth and development. He described it as:

… the urge which is evident in all organic and human life—to expand, extend, to become autonomous, develop, mature—the tendency to express and activate all the capacities of the organism, to the extent that such activation enhances the organism or the self (Rogers [32] p. 35).

Rogers saw actualizing progressing satisfactorily through childhood and into adult life provided the individual is able to grow in an atmosphere of “unconditional positive regard”, an experience of being valued as a person independently of whom or what they are. Under these circumstances the adult will be someone “comfortable in their own skin” and relatively resilient to the challenges of life. If instead the child grows in an atmosphere of conditional regard, one in which they experience themselves as only valued if certain conditions such as behaving well, working hard at school or containing emotions are met, then they will develop into adult life dependent upon having to fulfil such conditions if they are to feel good about themselves, and vulnerable to mental health difficulties if they are unable to. Rogerian therapy is conceived of as a relationship in which the client can experience unconditional positive regard and discover that there is no need to fulfil pre-set conditions before self- actualisation and personal growth can be realised.

Marie Jahoda made contributions to the tradition originally associated with the Mental Hygiene Movement. The National Committee for Mental Hygiene was founded in New York in 1909 by a number of leading psychiatrists and concerned others. It was commentary upon conditions in mental hospitals and attempts to improve them, but it was also concerned with “mental welfare”. In parallel with other approaches to hygiene this reflected an interest in preventive measures such as identifying and ameliorating emotional problems in young children, but it also gave birth to the idea of mental health as a positive state rather than merely the absence of “mental illness”. Jahoda was commissioned by the Joint Commission on Mental Illness and Health to summarise what was then established about “Positive Mental Health”. In that report she identified six dimensions of positive mental health or well-being. These were: attitudes of an individual towards his/her own self, the individual’s style of growth, development or self-actualisation, a quality described as integration or coherence of personality and which is related to the presences of a unifying outlook and resilience to stress, autonomy, a realistic relationship with context or external reality, and environmental mastery ([11] pp. 22–64). These clearly overlap with Maslow’s, and Rogers’ frameworks and remain the features most studied by more contemporary investigators and commentators [20, 34, 37].

## Identifying and Measuring Flourishing

Keyes’ [17] contribution has been to further clarify the distinction between psychological well-being and the absence of mental illness, and in doing so further emphasise the validity of a perspective upon well-being as a subjective quality distributed across a spectrum from flourishing to languishing.

In the course of the first MIDUS survey 3032 English speaking adults aged between 25 and 74 from 48 states completed self-report questionnaires exploring symptomatology that would or would not identify them as formally suffering from depression, rate their emotional well-being, their psychological well-being, their social well-being, provide a measure of psychosocial functioning and/or impairment and record a number of demographic variables [21].

Presence or absence of depression during the preceding 12 months was determined by reference to DSM-III-R diagnostic criteria [3]. Operationalised in the short form of the Composite International Diagnostic Interview (CIDI-SF, [15]).

Emotional well-being was rated on the basis of how much of the time during the last 30 days—“all”, “most”, “some”, a little” or “not at all”—respondents had felt “cheerful”, “in good spirits”, “extremely happy”, “calm and peaceful”, “satisfied” and “full of life”, and life satisfaction on a ten point scale ranging from “worst possible life overall “to “the best possible life overall”. Psychological well-being was rated on the basis of a measure which gauges how respondents see themselves thriving in their personal life over a set of six dimensions; self-acceptance, positive relations with others, personal growth, purpose in life, environmental mastery and autonomy [35]. Social well-being was rated on the basis of responses to a similarly constructed questionnaire exploring how well respondents saw themselves thriving in their social life across five dimensions; social acceptance, social actualisation, social contribution, social coherence, and social integration [16]. Psychosocial functioning and impairment were rated on the basis of evaluations of emotional, mental health as “poor”, “fair”, “good”, “very good” or “excellent” and the extent to which respondents considered their health limited them “a lot”, “some”, “a little” or “not at all” from doing any of nine activities of daily living such as bathing or dressing themselves, or vigorous exercise. Recorded socio-demographic variables included age, gender, race, marital status, employment status and educational attainment.

Consistent with the view that overall well-being is a complex emergent property reflecting interactions between emotional, psychological and social dimensions, scores on the four sets of well-being measures co-varied. There were only four cases where individuals scored high for psychological well-being but not for social well-being or one of the two measures of emotional well-being. There were only two cases where individuals returned a low score for psychological well-being but not for emotional or social well-being.

Individuals with scores in the upper tertiles of one of the two emotional well-being scales and six of the eleven individual measures of psychological and social well-being measures were classified as flourishing. Individuals with scores in the lower tertiles of one of the two emotional well-being scales and six of the eleven psychological and social well-being scales who were classified as languishing. The remainder were described as moderately mentally healthy.

On the basis of responses to the CIDI-SF 429 of the 3032 respondents were considered to have suffered an episode of depression in recent months. 143 of these were classed as languishing, 259 as moderately mentally healthy and 27 as flourishing. Of the 2603 who did not qualify for a diagnosis of “depression” only 520 (20 %) were considered to be flourishing and only 368 (14 %) were classed as languishing. χ^2^ calculated from these contingencies was significant (*p* < 0.001). Thus these data identify a dimension of psycho-social well-being which may overlap with the presence of formally identified mental illness but neither explains nor is explained by it. A significant proportion of those who were not formally “depressed” still reported a relatively low level of well-being; were languishing, and a small number of those who did qualify for having been depressed were nevertheless classified as flourishing.

When associations with disability were considered this dissociation between the importance of well-being and that of formally defined mental health status was even more striking. Comparable proportions of the languishing, the depressed but not languishing and the depressed and languishing reported “a lot” of limitation in one or more activities (64, 55 and 69 % respectively). This can be interpreted as evidence that that it is well-being rather than the presence of formally defined mental illness per se which limits activities. Regressions with socio-demographic variables indicated that higher levels of well-being and flourishing were associated with male gender, age (between 45 and 74), higher levels of education (16 or more years) and being married.

None of these findings are necessarily surprising but what Keyes et al. [18] have established in this and related publications, is that a meaningful construct can be quantified. It reflects more intuitive notions of well-being, it is not merely a proxy for the absence of symptoms otherwise identified as mental illness, and it is associated with capability, engagement and certain features of socio-demographic status. The ability to quantify well-being in this way has also made it possible to explore some of its determinants or antecedents, and one of the more energetically pursued lines of enquiry has been the relationship between well-being and the longer term effects of having survived a traumatic experience.

## Detecting and Measuring Post-traumatic Growth

A starting point for an account of recent posttraumatic growth research is follow-up studies of those involved in the Herald of Free Enterprise disaster. This was the abrupt capsizing of a cross channel ferry carrying some five hundred passengers and crew off Zeebrugge in March 1987. One hundred and ninety-three died and most of the survivors suffered profoundly traumatic experiences. Lawyers acting on behalf of the survivors and the bereaved contacted the Institute of Psychiatry in London seeking professional help, and a longer term follow-up study was born.

Initially the investigators were interested in determinants of subsequent psychological distress and at 3 years these were, most strongly, a sense of helplessness and loss of agency during the accident, poor subsequent social support and further adverse life events such as serious illness or unemployment. Some of the most distressed were those who continued to experience survivor guilt … concerning what they did not do to save others or did at others’ expense to save themselves. A significant proportion of them were using prescribed medication. On the whole they were not a pretty sight but there were some unexpected findings. When asked “Has your view of life changed since the disaster—and if so, has it changed in a positive way or a negative way?” 43 % said that their view of life had changed for the better. This was the beginning of a programme of research that has applied quantitative approaches to changes of outlook following trauma. On the basis of free text comments provided by those who had experienced positive changes following the Herald of Free Enterprise disaster Stephen Joseph and colleagues developed the Changes in Outlook Questionnaire (CiOQ). In its original form this was a 26 item questionnaire comprising fifteen negative statements such as “I no longer feel able to cope with things” and “I am less tolerant of others now”, and eleven positive statements such as “I live each day to the fullest now” and “I value other people more now”. Respondents were asked to endorse each item “strongly agree”, “disagree”, “disagree a little”, “agree a little”, “agree” or “strongly agree”. This questionnaire was used to investigate the condition of survivors following another maritime disaster, sinking of the cruise ship The Jupiter in Piraeus harbour on October 21st 1988. The percentage of adults endorsing agreement (of any degree) with the eleven positive statements ranged from 94 (I don’t take life for granted anymore) to 44 % (I don’t worry about death at all anymore) [13]. On the basis of factor extractions and other psychometrics the questionnaire has been refined to a ten item version [14], and these are given below:

1. I don’t look forward to the future anymore.
2. My life has no meaning anymore.
3. I don’t take life for granted anymore.
4. I value my relationships much more now.
5. I’m a more understanding and tolerant person now.
6. I no longer take people or things for granted.
7. I have very little trust in other people now.
8. I feel very much as if I’m in limbo.
9. I have very little trust in myself now.
10. I value other people more now.

As with the 26 item version respondents are asked to endorse each of these “strongly agree”, “disagree”, “disagree a little”, “agree a little”, “agree” or “strongly agree”. Other groups of investigators have devised their own similar scales. These include the Perceived Benefit Scale (McMillen and Fisher [24], the Posttraumatic Growth Inventory [36], the Stress Related Growth Scale [28] and the Thriving Scale [1]. A review of published uses of these to 2004 established rates of endorsing some positive items of posttraumatic growth of between 97 and 44 % amongst victims of traumatic experiences which included shipwreck, tornado, diagnosis of breast cancer, spinal injury and the development of chronic arthritis. The Posttraumatic Growth Inventory was the most widely used, and the only instrument providing data that could compare between experiences. Mothers bereaved of a child reported highest levels of posttraumatic growth and husbands of women with breast cancer the lowest. Cognitive appraisal variables (threat, harm, and controllability), problem-focus, acceptance and positive reinterpretation coping, optimism, religious practice, cognitive processing, and positive affect were all consistently associated with posttraumatic growth. There were inconsistent associations between posttraumatic growth and socio-demographic variables such as gender, age, education and income, and between posttraumatic growth and psychological distress variables such as depression, anxiety or a diagnosis of posttraumatic stress disorder. Predictably people who reported and maintained posttraumatic growth over time were less distressed [19].

## Associating Flourishing and Post-traumatic Growth

There is only limited empirical literature drawing together the phenomenon of posttraumatic growth, which this research identifies as an independent, valid, detectable and measurable phenomenon, and flourishing, which, on the basis of Keyes’ work, enjoys comparable ontological credentials. Furthermore, experiences of well-being associated with posttraumatic growth are very much those identified as “psychological” well-being, as opposed to those identified as “subjective” well-being. These are distinct [18]. The former refers to qualities such as autonomy, a sense of mastery, personal growth, positive relations with others, self-acceptance and purpose in life [34] whereas the latter refers to the balance between positive and negative feelings, life satisfaction and happiness. This clearly resonates with the classical distinction between Eudaimonism and Hedonism. Flourishing also resonates better with notions of “psychological” wellbeing and/or eudaimonism than with their counterparts. Thus it would be reasonable to predict, were the research to be conducted, that individuals who have enjoyed posttraumatic growth would also be found to have achieved a higher level of flourishing. An obvious reason for the shortage of such data is that it would be a very difficult set to obtain. It would require measurement of individuals’ location on the languishing—flourishing dimension before a traumatic event, and then again afterwards, along with other measures such as their scores on the Change in Outlook Questionnaire or the Posttraumatic Growth Inventory. This would involve recruiting subjects who were to undergo a predictably traumatic experience even before they were traumatised by the threat of it, and although major elective surgery might be a candidate trauma, ethical and logistic concerns make it very difficult to anticipate recruiting a sufficient number of subjects. Thus, for now at least, the association between posttraumatic growth and flourishing has to remain a highly probable assumption rather than an empirically derived “fact”. It receives some support from a study of the small proportion of a larger sample of undergraduate students who suffered a traumatic event between two responses to growth and well-being surveys conducted 8 weeks apart [7], but further research is needed to establish this as a more generalised empirical finding.

That alone should not prevent interest in what could be learned from assuming an association or even a causative relationship between the two phenomena. Another conclusion from Linley and Joseph’s [20] review was that higher levels of posttraumatic growth were associated with particular psychological orientations to experience of the event. Awareness and controllability of the traumatic experience were associated with better outcomes, as were acceptance, positive reinterpretation and positive religious coping. Similar findings have emerged from a meta-analysis seeking determinants of “benefit-finding” following trauma [10]. The term “benefit-finding” was used to refer to a wider range of developmental experiences following trauma than those captured by the narrowly quantitative questionnaire approaches considered by Linley and Joseph, but parallels are strong. Meta-analytic findings were that posttraumatic psychological benefit was negatively associated with depression, positively associated with positive well-being and, significantly, with higher levels of intrusive and avoidant thoughts concerning the event.

## Avoidance and Approach: Assimilation and Accommodation

It would appear that flourishing following a distressing experience is more closely associated with active engagement than with denial or psychological erasure. The distress of a traumatic event can be met with approach-oriented coping, such as addressing the situation and managing emotions, or with avoidance-oriented coping such as denial or the use of drugs and alcohol which suppress them. There is ample evidence that the former is more likely to lead to resolution and the latter to continuing difficulties ([12] pp. 117–135). Jean Piaget’s concepts of assimilation and accommodation [30] can be applied as a way of understanding this. In the course of observing children’s development Piaget developed the view that a psychological or behavioural repertoire could respond to newly encountered contingencies by assimilating them into the range of contingencies already covered by existing capabilities; an apple is a spherical object just like a ball, and so it is something to roll and play with, or by accommodating to new information by acquiring new repertoires of understanding and behaviour; an apple may be just like a ball in some ways but when you put it into your mouth and chew something nice comes out, and mother treats these particular balls in a different way from others, so there might be something to be gained by developing a new line on spherical objects. Psychological development has occurred. Traumatic experiences are, by definition challenges to pre-existing assumptions. Suddenly driving is no longer a relatively safe and mundane activity but one that can include painful and life threatening accidents. A secure future is abruptly undermined by the discovery of cancer. The world is literally turned upside down in the course of a shipwreck and the survivor experiences the agonies of those who do not survive. A cherished pregnancy comes to naught in the course of a miscarriage, or perhaps even worse, a stillbirth. All these and others challenge assumptions and demand a response. The experience is inevitably unpleasant and one reaction might be to deny its validity and press on regardless but that is unrealistic. The traumatic event has occurred and one person’s denial cannot speak for others. There may be undeniable consequences such as a broken limb or even a permanent disability. The reality of trauma is inescapable and an event which has touched others, altered perceptions of what the world is or resulted in clearly apparent disability cannot be assimilated into pre-existing routines of perception and behaviour. Routines of perception and behaviour have to change in order to accommodate the new. Growth occurs and life embarks upon a new course. If it doesn’t, the consequences of changed relationships, altered evaluations of safety and security and/or the effects of injury act as constraints upon the continuation of status quo ante.

## Growth Through Suffering

Leonard Cohen’s “…locked into your suffering and your pleasures are the seal” draws attention to much wider application of this principle. Reference has already been made to ways in which it is recognised in ancient teachings; in the Old Testament, perhaps most notably in the form of the Book of Job and early Greek distinctions between eudaimonic and hedonic experiences of well-being. Much of the Bhudda’s teaching concerns the psychological benefits of endurance and self-denial. One could argue that a central pillar of Christianity is the value of seeing beyond worldly suffering, and that is echoed in the Qur’an: “Be sure we shall test you with something of fear and hunger, some loss in goods or lives or the fruits (of your toil), but give glad tidings to those who patiently persevere” (Surah Al-Baqarah verse 155). More recent times see Viktor Frankl’s reflections upon experiences of life in Nazi concentration camps and subsequent lessons from patients as a result of which he maintained that confrontation with the “primordial facts” of existence offers the essential opportunity for finding meaning in life. Reference has made to Abraham Maslow’s study of self-actualization, which led him to conclude that the most important learning experiences in human life are tragedies, deaths and other traumas that force people to take new perspectives on life. Irwin Yalom has reflected:

A real confrontation with death usually causes one to question with real seriousness the goals and conduct of one’s life up to then … how many people have lamented: ‘What a pity I had to wait till now, when my body is riddled with cancer, to know how to live [38], p. 165).

There has to be no residual doubt that common human experiences include positive developments following significant distress or discomfort. These can be considered a form of growth, enlightenment, maturation, improvement, spiritual advancement or benefit-finding. They are distinct from simply adapting to the vicissitudes. They have more generalized consequences for and tend to be accompanied by wider effects upon well-being and relationships. Although the critical experiment has not been done and could be too difficult to arrange in a sufficiently convincing form, it is hard to escape the conclusion that these effects are indistinguishable from those also identified as flourishing.

## Current Times: Changes in What it Means to be “Ill”

Turning the scientific gaze upon these matters confirms them as phenomena meriting respect amongst those constrained by contemporary idioms, and helps direct thinking and research towards conventional preoccupations. Divine influence, self-actualization, or a search for meaning don’t cut much mustard in standard healthcare settings, but the notion of “recovery” does. Mental health policy and related services have become quite enamoured of approaches which advocate enhancing patients’ autonomy and self-management. The importance of enabling patients’ growth and development as an individual managing their own chronic condition in partnership with professionals rather than remaining a passive recipient of professional input is also respected in other areas of practice. What this might mean, the realistic probability of “recovery” from serious injury, can be illustrated by considering half a dozen previously fit young people who have all suffered a serious spinal injury leaving each of them irreversibly paraplegic. It is, surely, reasonable to suppose that after a couple of years the group will include one who has remained bed-bound and dependent, one who has become an aspiring para-athlete, and four who might be distributed across the spectrum between them. What has determined the differences between them is open to speculation. It isn’t the nature or severity of their injury; it will be a complex interaction of many social, personal and contextual variables of which we have little grasp, to date, but what is clear is that something of importance is at work, or isn’t, and it has a profound effect upon how well the individual has or hasn’t “recovered”; how successfully they have or have not flourished in the face of adversity.

In the context of contemporary healthcare policies and practices a successful outcome such as this is likely to be considered a fortunate accident rather than the result of comprehensible influences. Apposition of what is known about post-traumatic growth and flourishing suggests that the process can be understood, but recognising and employing its determinants presents a challenge to conventional frames of reference. Historically and into current times healthcare has been a predominantly paternalistic enterprise characterised by the application of formalised knowledge and skills by appropriately authorised and empowered practitioners. What is becoming clear about post-traumatic growth is that it is best understood as a manifestation of personal development and self-realisation. Rather than the result of this or that instrumental intervention on the part of a practitioner, it appears to be best understood as the outcome of an intrinsic propensity to make creative adjustment. As Rogers and others who have considered the role of the therapist in this process conclude, supportive, reliable and non-critical relationships can enable it, but they cannot be usefully considered to be instrumentally responsible for it [32]. That it happens at all is evidence of an essential but relatively overlooked dimension of humanity; a capacity, above all things, to make individual, creative and adaptive sense of the situation. Empirical evidence of this in the form of research findings that identify and confirm the nature and determinants of post-traumatic growth re-affirms the ontological stability of this phenomenon. This is a challenge to current approaches to healthcare. They are heavily framed by considerations of cost-effectiveness based upon scientifically derived evidence, and the activities of privileged practitioners. If optimal “healing” is to be the enabling of self-realisation, then what parts do science and professional interventions have to play, and what should healthcare commissioners consider themselves obliged to pay for?

These are not novel reflections. The rhetoric of person-centred-care is well established in professed healthcare policy (see, for instance the Health Care Foundation [9] and the [33] but there is considerable evidence that the goal of a truly patient-centred health care environment is proving hard to reach. Within the mental health field, where that goal is synonymous with attempting to enable “recovery” there is considerable enthusiasm for the ideal, but as much evidence that achieving it is not easy [6, 25, 31]. In a recently reported roundtable discussion participants agreed that “healthcare is about relationships” though also that it is “easy to pass the buck onto the clinicians and the professionals,” but if you do “you don’t have a partnership, and you don’t have a relationship unless both parties work at it.” [27]. Through her carefully conducted ethnography of diabetes care Annemarie Mol illustrates just the same point [26]. The institutionalised resistance to such changes is vividly illustrated in an anecdote provided by Don Berwick [4]:

Three years ago, a close friend began having chest pains. She headed for a cardiac catheterization, and, frightened, she asked me to go with her. As I stood next to her gurney in the pre-procedure room, she said, “I would feel so much better if you were with me in the cath lab.” I agreed immediately to go with her. The nurse didn’t agree.

Do you want to be there as a friend or as a doctor? she asked.

I guess both, I replied. I am both.

It’s not possible. We have a policy against that, she said.

The young procedural cardiologist appeared shortly afterward. I understand you want to have your friend in the procedure room, she said. Why?

Because I’d feel so much more comfortable, and, later on, he can explain things to me if I have questions, said my friend.

I’m sorry, said the cardiologist, I am just not comfortable with that. We don’t do that here. It doesn’t work.

Have you ever tried it? I asked.

No, she said.

Then how do you know it doesn’t work? I asked.

It’s just not possible, she answered. I am sorry if that upsets you.

Moments later, my friend was wheeled away, shaking in fear and sobbing.

Institutionalised healthcare continues to conform to a very rigid framework which can be considered a reflection of parameters moulded by the sick role. Parsons’ outline [29], pp. 428–479) has stood the test of time and remains an effective summary of this process. He identified a set of social arrangements that have evolved to accommodate the “social cost” of an ill person. Arrangements that include the application of specialised knowledge and skills are put in place to optimise the probability of recovery, the sick person is relieved of responsibilities for as long as they are formally identified as “ill”, and all concerned respect these rights and responsibilities as temporary, pending either recovery or death. This combination of structural and relational features provides a sufficient explanation for the form taken by most interactions between individuals and healthcare practitioners. It also provides a sufficient explanation for many of the institutional arrangements developed to provide it, including the privileged status of many healthcare practitioners and the priority given to the development of their skills and knowledge. Unfortunately it is a legacy from an era when the everyday nature of debilitating illness was different from that which it has become, in industrialised parts of the world, since the middle of the twentieth century.

Before the widespread introduction of sterile surgical techniques, effective antibiotics and immunisation during the second half of the nineteenth and the first half of the twentieth centuries, life-threatening illness commonly took the form of an acutely debilitating fever due to bacterial infection, such as pneumonia, puerperal sepsis or septicaemia from a gangrenous wound. Until very recently, common experience of illness was a fever that either ‘broke’—in other words, resolved as the body’s natural defences prevailed—or resulted in death. During the fever the victim would be incapacitated by pain and weakness, and personal hygiene, nutrition and fluid intake would have to be supported by others. Under these circumstances it would be adaptive to employ an institutionalised interaction between ‘patient’ and ‘carers’, in which the ‘patient’ temporarily surrenders autonomy in return for professional care and support which are realistically likely to improve their outcome, perhaps even save their life.

With the development of sterile surgical techniques and the availability of antibiotics, survival after serious injury such as spinal transection causing paralysis, loss of a limb or a brain-damaging head injury, have become much more likely than was earlier the case. Mortality following surgical amputation of a limb stood at around 60 % in the early nineteenth century. By 1910 it had fallen to some 10 % [2]. The need to accommodate the ‘disabled but no longer ill’ is a recent development that has only freshly found full expression in the form of disability rights legislation. Progress towards this occupied much of the last century. It includes redeeming the disabled from a status of ‘patronised and dependent person’ that is characteristic of a patient inhabiting the classic sick role, to that of ‘autonomous and independent person’ with full expectations of rights and responsibilities. This is the territory of truly patient-centred practice; a collaborative relationship in which the practitioner enables the “patient” to realise themselves in their new and changed circumstances. Technology and specific skills might be involved but the relationship is a partnership rather than one between client and provider. Full implications of this for healthcare practitioners and their patients are only just beginning to be realised. On the one hand practitioners have to forgo the status of authoritative expert and accept a more facilitating role, and on the other “patients” have to accept their difficulties and own the task of recovering or accommodating to persistent disability. These are very much the lessons that are being learned from studies of post-traumatic growth and, by analogy, that owning and accommodating to difficulties enables flourishing far more effectively than handing them over to someone else to “treat”. They are challenging changes for centuries-old traditions of expectation, practice, training and institutional arrangement. Furthermore ancient wisdoms largely enshrined in religious teaching date from pre-antibiotic times when the classic sick role was more appropriately applicable. As a result the philanthropy they also embody makes a distinction between the propriety of supportive paternalism in response to “illness” and the value of redemption through suffering by other cause. Changes in the nature of “illness” occasioned by little more than a 100 years’ insight into bacterial infections and how to treat or prevent them has blurred that distinction. Fully embracing the value of redemptive suffering might have equally wide ranging consequences.

Cohen’s 1960s countercultural comments reflected wider dissatisfactions with consumerism and symbolic attractions that he and his contemporaries railed against. Despite their efforts the intervening half century has not seen a significant change in cultural trajectory. Situated as it is on the boundary between natural and human worlds, healthcare can and has functioned as an anvil upon which collective misunderstandings are hammered out. Changes in our understanding of the nature and significance of micro-organisms were forged in Lister’s operating theatre but they have had much wider influence. Recognition of the harm caused by early separation of child from parent began with Bowlby’s observations of children in care [5], but the wider implications of recognising children’s needs for care and emotional stability extend far much wider. Keyes’ work identifies flourishing, an optimal state of mind, in less than a fifth of the population. Studies of posttraumatic growth and the dynamics of recovery born out of healthcare encounters suggest that creative confrontation with adversity engenders flourishing, as many ancient wisdoms argue. In addition to healthcare, how well are our wider economic structures and tokens of esteem aligned to this?

## Conclusion

Recent empirical research has established a robust measure of flourishing, and that this is only identifiable in a proportion of sampled populations. Parallel but separate empirical research has established that a significant proportion of those suffering a traumatic experience enjoy post-traumatic growth and personal development not unlike flourishing. The determinants of this experience appear to have a lot in common with ancient wisdoms extolling the virtues of suffering as a route to spiritual and/or psychological development.

This has direct relevance in the context of healthcare. Many of the roles, expectations and institutional arrangements it embodies reflect application of the orthodox sick role, which serves to insulate against suffering. Historically very recent changes in the nature of illness make these outmoded. Lessons from the study of flourishing and post-traumatic growth might inform alternatives. These lessons endorse the ontological stability of phenomena linked to the longer term benefits of suffering, and suggest that healthcare could indeed benefit from becoming the more collaborative enterprise encouraged by the ideals of patient-centred care. The deeply embedded orthodoxies of the sick role and its professionalised concomitants can be seen as hindrances to this. Furthermore, recognition of post-traumatic growth as a route to flourishing has wider implications for a society increasingly attached to convenience and immediate gratification.
